# Time Dependent Gene Expression Changes in the Liver of Mice Treated with Benzene

**DOI:** 10.4137/bmi.s590

**Published:** 2008-03-28

**Authors:** Han-Jin Park, Jung Hwa Oh, Seokjoo Yoon, S.V.S. Rana

**Affiliations:** 1 Korea Institute of Toxicology, 100-Jang-Dong, Yuseong-Gu, Daejeon-305-600, Korea; 2 Toxicology Laboratory, Department of Zoology, Ch. Charan Singh University, Meerut (India) – 250004

**Keywords:** benzene, liver, gene expression, circadian rhythms, molecular markers, pathways

## Abstract

Benzene is used as a general purpose solvent. Benzene metabolism starts from phenol and ends with p-benzoquinone and o-benzoquinone. Liver injury inducted by benzene still remains a toxicologic problem. Tumor related genes and immune responsive genes have been studied in patients suffering from benzene exposure. However, gene expression profiles and pathways related to its hepatotoxicity are not known. This study reports the results obtained in the liver of BALB/C mice (SLC, Inc., Japan) administered 0.05 ml/100 g body weight of 2% benzene for six days. Serum, ALT, AST and ALP were determined using automated analyzer (Fuji., Japan). Histopathological observations were made to support gene expression data. *c*-DNA microarray analyses were performed using Affymetrix Gene-chip system. After six days of benzene exposure, **twenty five genes** were down regulated whereas **nineteen genes** were up-regulated. These gene expression changes were found to be related to pathways of biotransformation, detoxification, apoptosis, oxidative stress and cell cycle. It has been shown for the first time that genes corresponding to circadian rhythms are affected by benzene. Results suggest that gene expression profile might serve as potential biomarkers of hepatotoxicity during benzene exposure.

## Introduction

Benzene is widely used as a general purpose industrial solvent. However, it is now used principally in the synthesis of other chemicals. Its toxic effects on the blood and bone marrow include leucopenia, pancytopenia, aplastic anemia and leukemia (Snyder, 2002). Several studies on benzene have focused on its metabolic pathways to determine its hematotoxicity and leukemogenicity (Henderson, 1996; Snyder and Hedli, 1996). Metabolism of benzene to reactive metabolites by hepatic enzymes mainly cytochrome P_450_2E1 (CYP2E1) is a prerequisite to its cyto and genotoxicity (Gut et al. 1996; Valentine et al. 1996). Primary benzene metabolites include phenol, hydroquinone, catechol and trans trans muconic acid (Ross, 2000). They interact synergistically and further exacerbate benzene toxicity (Chen and Eastmond, 1995). This mechanism of multimetabolite genotoxicity is another unique aspect of benzene that distinguishes it from other chemicals in terms of mechanism of its toxicity. Subsequent secondary activation of its metabolites by myeloperoxidase (MPO) present in bone marrow results in the production of genotoxic quinines and reactive oxygen species. They induce hemopoetic cellular damage and dysfunction of bone marrow stromal cells (Farris et al. 1997). Despite intensive studies over several decades, mechanisms underlying benzene induced toxicity and leukemogenicity are not yet fully understood. They are complicated by various pathways including those of metabolism (Snyder and Hedli, 1996), growth factor regulation (Niculescu, 1995), oxidative stress (Laskin et al. 1996), DNA damage (Lee and Garner, 1991), cell cycle regulation (Yoon et al. 2001b) and programmed cell death (Ross et al. 1996). To elucidate molecular mechanisms of its toxicity, cDNA microarray analyses have been performed by several workers. Yoon et al. (2003) performed cDNA microarray analysis to study the mechanisms of benzene induced hematotoxicity in bone marrow of mouse. Effects of oral exposure to benzene on gene expression in rat liver were reported by Heijne et al. (2005). Gene expression changes were attributed to pathways of biotransformation viz. glutathione synthesis, fatty acid and cholesterol metabolism. Kim et al. (2005) suggested that hydroquinone at least in part, may enhance allergic immune responses of benzene by inhibiting the production of IL-12 in macrophages. Microarray analysis of peripheral blood of benzene exposed workers was performed by Forrest et al. (2005). They showed that the altered expression of CXCL16, ZNF331, JUN and PF4 as potential biomarkers of benzene exposure. Tumor related (Xia et al. 2005) and immune responsive genes (Wang et al. 2005) have also been recorded in patients suffering from benzene poisoning.

Since global gene expression studies are now being increasingly used in toxicology (Waters, et al. 2003), we hypothesized that microarrays could identify changes in gene expression in the liver that could be used as new biomarkers of exposure, identify early effects and provide information on mechanism of benzene toxicity. In this study we investigated the changes in gene expression induced by 24 hrs and six days of exposure to benzene in the liver of mice to probe further molecular mechanisms of benzene toxicity. The overall objective of the study is to identify potential molecular markers of early effects of benzene in liver.

## Materials and Methods

### Chemicals

Benzene was purchased from Sigma-Aldrich (U.S.A.). Trizol was purchased from Invitrogen (U.S.A.). RNA isolation kit was procured from Qiagen (Germany).

### Animals and treatments

Approximately 9-week old, male BALB/c mice (SLC, Inc., Japan) were kept in a 12 h light/dark cycle under controlled temperature and humidity for 2 weeks in the animal room prior to the experiment. The mice were offered standard food pellets and water ad libitum. Their body weight averaged from 24 to 27 g (mean ± S.D. −25.75 ± 0.97).

Pure benzene (99%) was diluted by corn oil so as to make a 2% (v/v) solution of benzene in corn oil. 0.05 ml of diluted benzene/100 g body weight was intraperitoneally injected to mice. 5 benzene treated mice were sacrificed after 24 h of benzene treatment. While 5 were sacrificed after 6 days of benzene treatment. Parallel controls were also sacrificed simultaneously.

### Biochemical analysis

Blood was collected from the inferior vena cava. Serum was separated by centrifugation. Serum activities of alanine aminotransferase (ALT), aspartate aminotransferase (AST) and alkaline phosphatase (ALP) were determined using automated Clinical Chemistry Analyzer (Fuji Dri-Chem 3500s, Fujifilm Co., Japan) at each time point. Data was statistically calculated and significance difference between treated and control groups was tested using one way analysis of variance (ANOVA) followed by the Dunnett test. P values less than 0.05 were considered to be significant.

### Histopathology

Small pieces of liver collected from the mid liver lobe of all treated and control mice were fixed in 10% neutral buffered formalin, dehydrated in alcohol, cleared in xylene and embedded in paraffin. Four microns thick paraffin sections thus prepared using rotary microtome (2165, Leica, Germany) were stained with hematoxylin and eosin and examined under light microscope (E400, Nikon, Japan).

### Isolation of RNA

Liver samples were homogenized with 2 ml Trizol^®^ (Invitrogen, U.S.A.) and mixed with 0.4 ml chloroform. Following centrifugation, aqueous phase was collected and mixed with isopropanol for precipitation. RNA precipitate was washed by ethanol. Isolated total RNA was purified using RNeasy mini kit (Qiagen, Germany) according to the manufacturer’s protocol. The total RNA was quantified using NanoDrop^®^ ND-1000 (NanoDrop, U.S.A.) and RNA integrity was determined by Agilent Bioanalyzer 2100 (Agient Technologies, U.S.A.).

### Microarray analysis

Affymetrix GeneChip^®^ Mouse 430A 2.0 containing over 22,600 probe sets was used for microarray experiment. Double-stranded cDNA was synthesized from 5 ug of template total RNA using One-Cycle cDNA Synthesis kit (Affymetrix, U.S.A.), and biotin labeled cRNA was synthesized using IVT Labeling kit (Affymetrix, U.S.A.). The labeled cRNA was purified with GeneChip^®^ Sample Cleanup Module (Affymetrix, U.S.A.). The quality and quantity of the cRNA was checked by conducting gel electrophoresis and NanoDrop^®^ ND-1000 (NanoDrop, U.S.A.), respectively. Fifteen micrograms of the purified cRNA of each sample was fragmented and hybridized to arrays for 16 h at 45 °C. All arrays were washed and stained automatically by using a fluidics Station 450 (Affymetrix, U.S.A.) and scanned by GeneChip^®^ scanner 3000 (Affymetrix, Inc, U.S.A.). All procedures were performed according to the manufacturer’s protocols (Affymetrix, U.S.A.). Image processing was performed using Affymetrix GeneChip^®^ Operating System (GCOS).

### Gene expression analysis

The preprocessing procedure of resultant cell intensity files (CEL) and following statistical analysis were performed using GenePlex software version 2.3 (ISTECH Inc., Korea). Microarray data was globally normalized and used for further analysis. Total 22,600 probe sets representing over 14,000 mouse genes were applied to gene expression analysis as well as hierarchical clustering. The differentially expressed genes were selected at a minimum 2-fold change after 1 day and 6 days of benzene treatment compared with the control group. Data were statistically analyzed by Student’s t-test and *P*-values <0.01 were considered as statistically significant. The selected genes were filtered by extension GCOS filtering (reference M1). In addition, the differentially expressed genes at 6 days treatment group compared with 1day treatment group were also selected considering the same criteria (≥2-fold, *P* < 0.01). All selected genes were analyzed by two-dimensional hierarchical clustering based on Pearson correlation and Complete Linkage (Eisen, 1998). Discrepancies among control, 1 day, and 6 days group were visualized by Principal Component Analysis (PCA). The classification of pathway for interesting genes was performed using KEGG pathway database. The selected genes were annotated based on NetAffx, linked at http://www.affymetrix.com.

## Results

### Blood biochemistry

**Figure 1:**
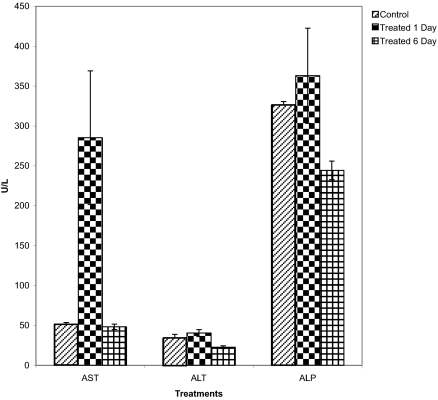
Serum levels of aspartate amino transferase (AST), alanine amino transferase (ALT) and alkaline phosphatase (ALP) after treatment with benzene at two time points i.e. one day and six days.

Aspartate aminotransferase (AST) and alanine aminotransferase (ALT) values increased significantly after 24 hours of benzene treatment. However, on treatment for 6 days, a significant decline in their activity was recorded. Activity of serum alkaline phosphatase also increased after 24 hours but declined after six days of benzene treatment (Fig. 1).

### Liver histopathology

**Figure 2:**
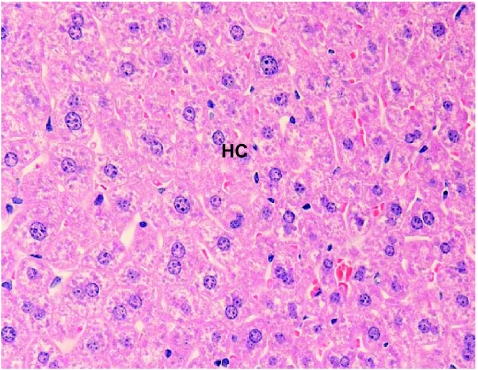
Normal hepatocytes without any atrophy or hypertrophy were observed in the liver of control mice. ×400.

**Figure 3:**
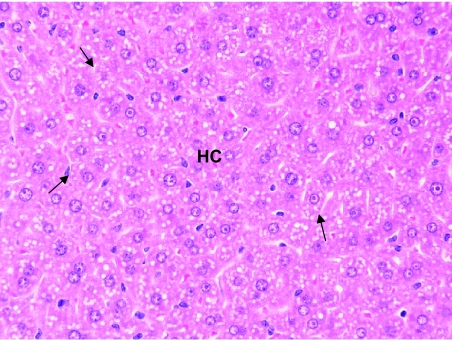
T.S. of the liver of a mice treated with benzene for 24 hrs shows microbalooning of the hepatocytes (HC). A few binucleated cells are also observed. ×400.

**Figure 4:**
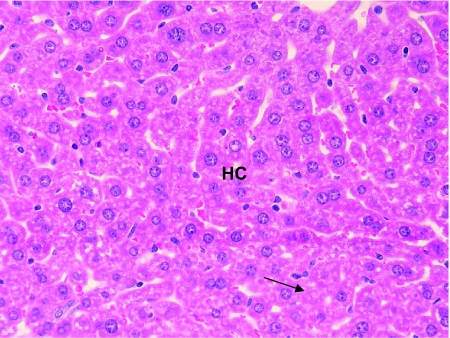
T.S. of the liver of mice treated with benzene for 6 days shows no microbalooning of the hepatocytes and no paranchymal degradation. However, mild hypertrophy was observed at certain places. Enlarged nuclei are also observed. ×400.

Hepatocytes near the centrilobular regions of the lobule were compared in all the three groups. No significant histopathological changes were observed in the liver of mice treated with benzene for 6 days. However, hypertrophic lesions were recorded at a few places (Fig. 4). However, microbalooning in the hepatocytes was observed in the liver of mice after 24 hr of benzene treatment. Large necrotic spaces were wanting (Fig. 3). No pathological lesions were observed in the liver of control mice (Fig. 2).

### Gene expression profile

Microarray analysis was carried out to determine differences in hepatic gene expression between benzene and vehicle (corn oil) treated mice. RNA integrity was intact (data not shown). Individual liver samples from treated animals were analysed. Array based observations were made in triplicate. Gene expression was analyzed only in animals treated with benzene for 24 hours and 6 days.

**Figure 5:**
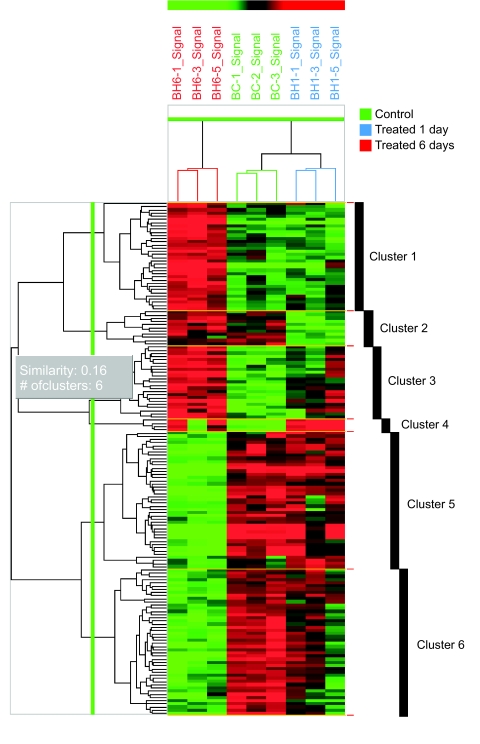
Hierarchical clustering map of gene expression profiles of all selected genes at the statistical criteria of ≥2-fold change and *p* < 0.01. Identification of genes that can differentiate between 6 days and 1 day groups after benzene treatment (The gene expression pattern of control group shows similar to 1 day group after benzene treatment). Each row represents a gene on the microarray and each column represents an individual hepatic mRNA sample of mice. The red and green indicate increased and decreased expression relative to the control, respectively. The colored branch on each column indicates each sample group: control (Green), 1 day (blue) and 6 days (red) group after benzene treatment.

A total of 136 reliable genes were filtered from 22600 probe sets by standard deviation of control strength after per spot and per chip normalization. Genes were sorted according to Student’s “t” test (*P* values from 0 to <0.05). Forty four genes were selected based on the fold difference in at least three of nine conditions using ANOVA. A summary of these analyses is presented in table 1. Table 2 lists genes that changed significantly (p < 0.05→0.01) in mice liver treated with benzene for six consecutive days. Results exhibiting time dependent changes in the expression of genes corresponding to control mice are explained in Figure 5.

**Figure 6:**
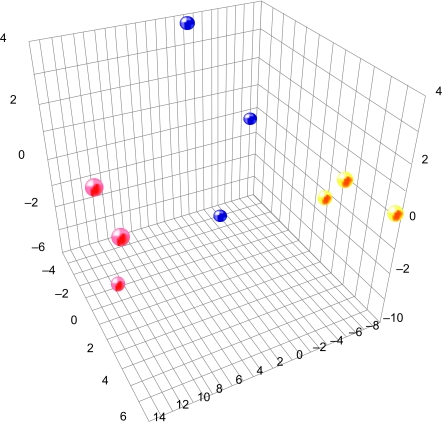
Principal component analysis shows gene expression changes of 136 select genes at varying time-points. The colored circles represent tested groups as follows: control, Blue; 1 day group after benzene treatment, Yellow; 6 days group after benzene treatment.

Hierarchical clustering of the genes and duration of treatment with benzene also revealed time dependent differences in gene expression (Fig. 5). Figure 6 exhibits principal component analysis showing gene expression changes.

Interestingly, proteins involved in biosynthesis of steroids (Hmger), cell cycle (Mcm5 and Mee), fatty and biosynthesis (Fasn), glutathione metabolism (Gstm3), glycerolipid metabolism (Lipg), glycolysis (Pklr), gluconeogenesis, MAPK signaling pathways (Hspa1a) and purine metabolism (Ecgf1) were found to be induced by benzene.

However, genes responsible for metabolism of xenobiotics (Cyp2b10), transcription factor (Gtf2ird1), cell adhesion (Cldn15 and Cldn1), circadian rhythm (Per3 and Arnt1), cytokines (Pdgfc), and glycine, serine and thireonine metabolism (Gldc) were down regulated (Table 1).

Important pathways affected after 24 hrs of benzene treatment included β-cell receptor signaling pathways (Ifitm1), cell cycle; signaling pathway; focal adhesion; Jak-STAT signaling pathway, circadian rthym (Cry1), cytokine-cytokine receptor interaction (Ccr5), glutathione metabolism (Gstm3), hematopoietic cell lineage (Cd24a) and TGF-beta signaling pathway (Id3 and Id1).

### Analysis of pathway level

After t test and fold differences filtering, 44 genes were found to be up or down regulated by benzene treatment. Analysis of differentially regulated genes showed the involvement of calcium signaling pathway, cell adhesion molecules pathway, circadian rhythms, glutathione-S-transferases and heat shock proteins in benzene toxicity.

## Discussion

It has been accepted now that omic technologies have significant potential in generating novel biomarkers of exposure, susceptibility and response to different xenobiotics. Different xenobiotics produce specific gene expression patterns in target organs. Minami et al. (2005) in their study on acetaminophen (APAP), bromobenzene, carbontetrachloride, dimethylnitrosamine and thioacetamide identified potential biomarkers of their hepatotoxicity. However, gene expression changes induced by benzene in the liver are yet to be established. Smith et al. (2005) showed that toxicogenomics have significant potential in generating biomarkers of benzene toxicity. They identified more than 100 differentially expressed genes, those related to apoptosis and immune function were most significantly affected in humans occupationally exposed to benzene. Yoon et al. (2003) investigated benzene induced hematotoxicity and leukemogenicity using mouse bone marrow and making cDNA microarray analysis in C57BL/6, wild type (WT), and p53-knock out (KO) mice. They suggested that dysfunction of p53 gene induced fatal problems finally resulting in hemopoietic malignancies.

Benzene induced gene expression changes in rat liver after 28 days of oral exposure to benzene showed disturbances in pathways of biotransformation, glutathione synthesis, fatty acid and cholesterol metabolism (Heijne et al. 2005). Wang et al. (2005) in their study on peripheral white blood cell gene expression profile of benzene poisoned patients identified 38 genes to be associated with benzene toxicity. Chen et al. (2005) made cDNA microarray analysis of leukocytes in patients of benzene poisoning and found 25 genes to be differentially expressed. Amongst these 16 genes were up-regulated and 9 genes were down regulated. Some cDNA replication and some repair genes associated with benzene poisoning also showed differential expression.

Time and dose dependent study on gene expression made by us shows that after six days of benzene treatment, 44 genes were affected. 25 genes were down regulated and 19 genes were upregulated. This information was further analysed through KEGG pathway database. Important pathways affected after 24 hrs of benzene treatment included β-cell receptor signaling pathways (Ifitm1), cell cycle; signaling pathway; focal adhesion; Jak-STAT signaling pathway, circadian rthym (Cry1), cytokine-cytokine receptor interaction (Ccr5), glutathione metabolism (Gstm3), hematopoietic cell lineage (Cd24a) and TGF-beta signaling pathway (Id3 and Id1).

We for the first time report that circadian rhythm pathway (Cry1) is also affected by benzene treatment. Rana et al. (2007) observed that circadian rhythms effect lipid peroxidation induced by benzene. Susceptibility of humans and rats to benzene during morning and evening was conducted by Leung and Harrison (1998) and Starek et al. (1990). Down regulation of Per3 and Arnt7 observed after six days of benzene treatment clearly shows the influence of circadian rhythms on benzene toxicity. Circadian rhythms are further regulated by protein synthesis and we show that these genes too were influenced by benzene. Present report confirms the influence of circadian rhythms on benzene hepatotoxicity. Rana and Verma (2005) reviewed the biochemical toxicity of benzene. However, this new information is expected to help in understanding the molecular mechanisms of benzene toxicity.

Interestingly, proteins involved in biosynthesis of steroids (Hmger), cell cycle (Mcm5 and Mee), fatty acid biosynthesis (Fasn), glutathione metabolism (Gstm3), glycerolipid metabolism (Lipg), glycolysis (Pklr), gluconeogenesis, MAPK signaling pathways (Hspa1a) and purine metabolism (Ecgf1) were found to be induced by benzene. However, genes responsible for metabolism of xenobiotics (Cyp2b10), transcription factor (Gtf2ird1), cell adhesion (Cldn15 and Cldn1), circadian rhythm (Per3 and Arnt1), cytokines (Pdgfc), and glycine, serine and thireonine metabolism (Gldc) were down regulated (Table 1).

Biochemical and histopathological observations show that six days treatment with benzene encouraged an adaptive mechanism in the liver. Gene expression studies especially those of CYP2610, CYP4A10 that were found to be down-regulated find support from the biochemical and histopathological results. Upregulation of Gstm3 and Hspa1a further suggest a protective mechanism.

The pathway analysis shows the involvement of calcium signaling, transferases and heat shock proteins in benzene toxicity. Present observations offer additional information on the molecular mechanisms of benzene toxicity. The cDNA microarray system showed alterations in the number of benzene affected genes including physiologic and toxicologic gene repertoires. This communication provides valuable targets for future investigations on the benzene induced toxicity and leukernogenicity.

## Figures and Tables

**Table 1:** Gene expression profile in the liver of mice affected by 6 days of benzene treatment.

Common name	Description	Average fold change	P-Value	mRNA accession no
**DOWN REGULATED**				
Cyp2b10	cytochrome P450, family 2, subfamily b, polypeptide 10	0.281536405	0.001011593	NM_009998; NM_009999
Gtf2ird1	general transcription factor III repeat domain-containing 1	0.413325939	0.004871763	NM_020331
Avpr1a	arginine vasopressin receptor 1A	0.324488832	0.004461284	NM_016847
Avpr1a	arginine vasopressin receptor 1A	0.36705761	0.001599135	NM_016847
H2-Aa	histocompatibility 2, class II antigen A, alpha	0.306047979	0.004596735	NM_010378
Cldn15	claudin 15	0.48776276	2.72E-04	NM_021719
Cldn1	claudin 1	0.260820687	5.28E-04	NM_016674
Cldn1	claudin 1	0.229626388	0.002089479	NM_016674
Clock	circadian locomoter output cycles kaput	0.377440404	5.77E-05	NM_007715
Clock	circadian locomoter output cycles kaput	0.424112305	0.004717732	NM_007715
Arntl	aryl hydrocarbon receptor nuclear translocator-like	0.173296253	0.001789661	NM_007489
Pdgfc	platelet-derived growth factor, C polypeptide	0.344610509	9.98E-04	NM_019971
Cyp4a10; BC013476	cytochrome P450, family 4, subfamily a, polypeptide 10; cDNA sequence BC013476	0.065019519	5.66E-04	NM_010011; NM_201640
Pla2g7	phospholipase A2, group VII (platelet-activating factor acetylhydrolase, plasma)	0.453285144	5.67E-04	NM_013737
Gldc	glycine decarboxylase	0.422548113	0.001410781	NM_138595
Chka	Choline kinase alpha	0.313032262	0.001080842	NM_001025566; NM_013490
Cd24a	CD24a antigen	0.395079154	0.003244021	NM_009846
Ddc	dopa decarboxylase	0.403510366	0.001461128	NM_016672
Psen2	presenilin 2	0.480851803	0.001028252	NM_011183
Mthfr	5,10-methylenetetrahydro-folate reductase	0.312663634	6.40E-04	NM_010840
Hmox1	heme oxygenase (decycling) 1	0.442942648	0.008907761	NM_010442
Ecgf1	endothelial cell growth factor 1 (platelet-derived)	0.45767403	0.001281208	NM_138302
Sphk2	sphingosine kinase 2	0.449968706	5.78E-04	NM_020011; NM_203280
Tjp3	tight junction protein 3	0.328536042	0.003973921	NM_013769
Tjp2	tight junction protein 2	0.494447722	0.001988285	NM_011597
**UP REGULATED**				
Hmgcr	3-hydroxy-3-methylglutaryl-Coenzyme A reductase	2.891840187	0.001640616	NM_008255
Aacs	acetoacetyl-CoA synthetase	2.563580176	0.003499423	NM_030210
Mcm5	minichromosome maintenance deficient 5, cell division cycle 46 (S. cerevisiae)	2.300496316	0.003483556	NM_008566
Wee1	wee 1 homolog (S. pombe)	7.053930707	0.001572758	NM_009516
Per2	period homolog 2 (Drosophila)	3.369578754	0.008408468	NM_011066
Bhlhb2	basic helix-loop-helix domain containing, class B2	3.728728789	0.001394815	NM_011498
Per3	period homolog 3 (Drosophila)	3.854051492	0.008180266	NM_011067
Fasn	fatty acid synthase	2.499244207	0.001200169	NM_007988
Gstm3	glutathione S-transferase, mu 3	3.142999858	0.00703031	NM_010359
Lipg	lipase, endothelial	2.670982122	5.59E-04	NM_010720
Pklr	pyruvate kinase liver and red blood cell	2.80962902	0.001207647	NM_013631
Pklr	pyruvate kinase liver and red blood cell	2.946723297	0.006457597	NM_013631
Pklr	pyruvate kinase liver and red blood cell	2.922004272	3.51E-04	NM_013631
Hspa1a	heat shock protein 1A	8.965401248	0.00735716	NM_010479
Egfr	epidermal growth factor receptor	2.884090081	0.005691373	NM_007912; NM_207655
Gadd45a	growth arrest and DNA-damage-inducible 45 alpha	2.007231563	0.008967359	NM_007836
Ppat	phosphoribosyl pyrophosphate amidotransferase	2.204661449	0.001375383	XM_001002879; XM_001002886; XM_896000; XM_924520; XM_973896; XM_973937; XM_973938; XM_973973
Gbe1	glucan (1,4-alpha-), branching enzyme 1	2.143906958	0.002461176	NM_028803
Gys2	glycogen synthase 2	2.108060527	0.002009201	NM_145572

**Table 2:** Comparison of gene expression profile in the liver of mice between two time points (One day treatment and six days treatment).

Common name	Description	Average fold change	P-Value	mRNA accession no
P4ha1	procollagen-proline, 2-oxoglutarate 4-dioxygenase (proline 4-hydroxylase), alpha 1 polypeptide	2.591853506	0.004172616	NM_011030
P4ha1	procollagen-proline, 2-oxoglutarate 4-dioxygenase (proline 4-hydroxylase), alpha 1 polypeptide	2.104156061	0.006374143	NM_011030
Ifitm1	interferon induced transmembrane protein 1	2.049667285	1.57E-04	NM_026820
Ccnd2	cyclin D2	2.296233152	0.005968229	NM_009829
Clock	circadian locomoter output cycles kaput	0.395914404	8.26E-04	NM_007715
Cry1	cryptochrome 1 (photolyase-like)	2.016943891	0.005553137	NM_007771
Acly	ATP citrate lyase	2.0650066	0.001685516	NM_134037
Ccr5	chemokine (C-C motif) receptor 5	0.448882656	0.007355149	NM_009917
Fasn	fatty acid synthase	2.612494125	4.47E-04	NM_007988
Cyp4a14	cytochrome P450, family 4, subfamily a, polypeptide 14	0.041921708	0.004694101	NM_007822
Cyp4a10;	cytochrome P450, family 4, subfamily	0.064562689	0.001113474	NM_010011;
BC013476	a, polypeptide 10; cDNA sequence BC013476			NM_201640
Gstm3	glutathione S-transferase, mu 3	3.251600332	0.005704424	NM_010359
Pla2g7	phospholipase A2, group VII (platelet-activating factor acetylhydrolase, plasma)	0.451785594	0.001250882	NM_013737
Pklr	pyruvate kinase liver and red blood cell	2.600750828	0.001583053	NM_013631
Pklr	pyruvate kinase liver and red blood cell	3.252169263	0.00849695	NM_013631
Pklr	pyruvate kinase liver and red blood cell	2.753805631	0.003288804	NM_013631
Cd24a	CD24a antigen	0.444671589	0.003072713	NM_009846
Hspa1a	heat shock protein 1A	8.966809881	0.006796064	NM_010479
Hspb1	heat shock protein 1	2.349998728	0.008158746	NM_013560
Id3	inhibitor of DNA binding 3	2.42776977	2.65E-04	NM_008321
Id1	inhibitor of DNA binding 1	2.226588424	1.97E-04	NM_010495
